# Supplementary material for: The essential calcium channel of sperm CatSper is temperature-gated
Source: Nat Commun. 2025 Apr 17;16:3657. doi: 10.1038/s41467-025-58824-0 (PMC12006431; doi:10.1038/s41467-025-58824-0)
Supplement: Supplementary file 1 — Supplementary Information [file 41467_2025_58824_MOESM1_ESM.pdf]

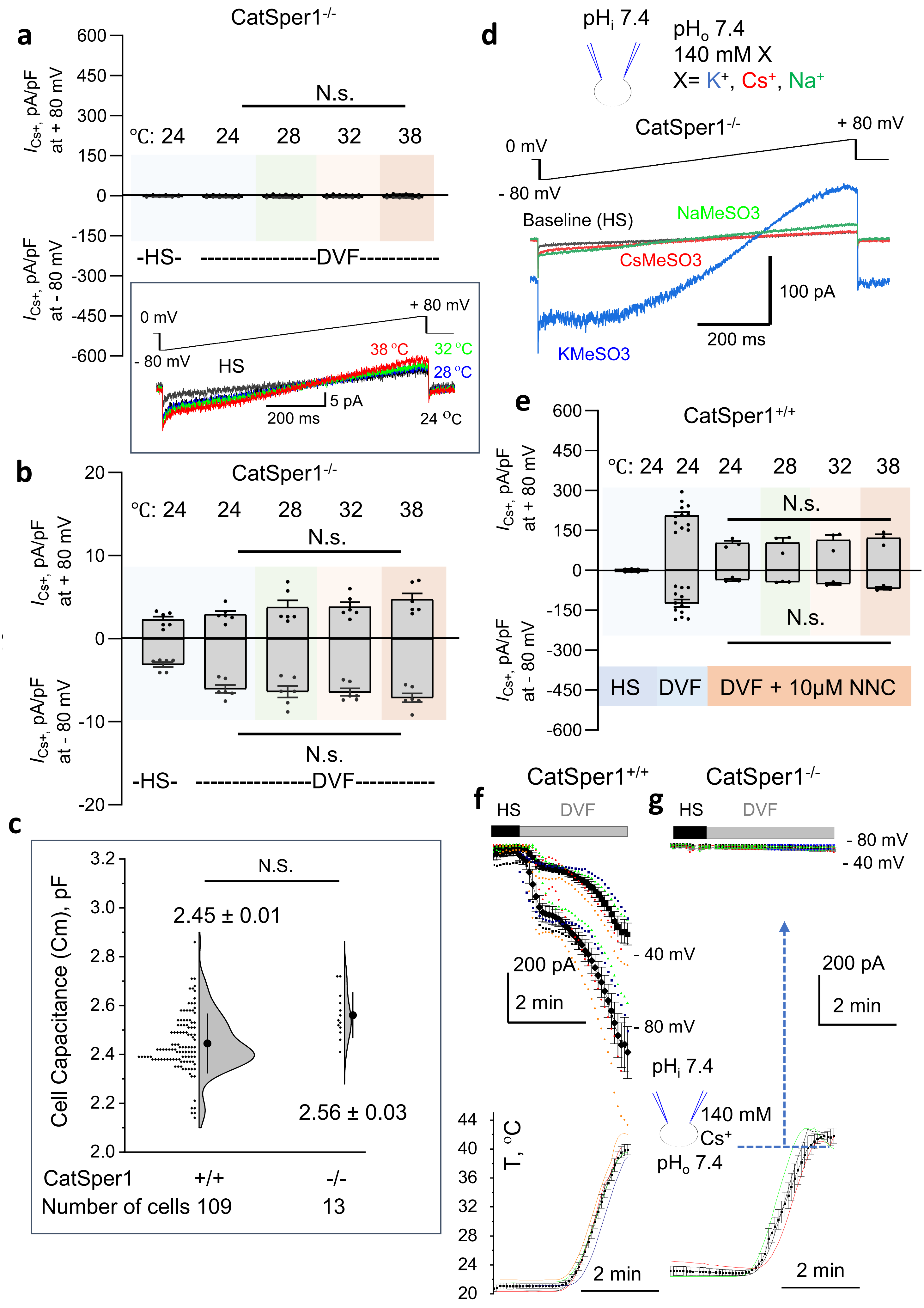

a
CatSper1-/-
I_{Cs+}, pA/pF at + 80 mV
I_{Cs+}, pA/pF at - 80 mV
N.s.
°C: 24 24 28 32 38
-HS- ----------------DVF-----------
0 mV
+ 80 mV
- 80 mV
HS
38 °C
32 °C
28 °C
5 pA
200 ms
24 °C
b
CatSper1-/-
°C: 24 24 28 32 38
N.s.
N.s.
-HS- ----------------DVF-----------
c
Cell Capacitance (Cm), pF
N.S.
2.45 ± 0.01
2.56 ± 0.03
CatSper1 +/+ -/-
Number of cells 109 13
d
pHi 7.4
pHo 7.4
140 mM X
X= K+, Cs+, Na+
CatSper1-/-
0 mV
+ 80 mV
- 80 mV
Baseline (HS)
NaMeSO3
CsMeSO3
100 pA
200 ms
KMeSO3
e
CatSper1+/+
°C: 24 24 24 28 32 38
N.s.
N.s.
HS DVF DVF + 10μM NNC
f
CatSper1+/+
HS DVF
- 40 mV
200 pA
2 min
- 80 mV
g
CatSper1-/-
HS DVF
- 80 mV
- 40 mV
200 pA
2 min
pHi 7.4
140 mM Cs+
pHo 7.4
T, °C
2 min
2 min

Supplementary Fig. 1 Murine sperm CatSper is a heat-activated channel. **a** Non-CatSper I_{Cs+} densities (pA/pF) stimulated by voltage ramps and recorded from CatSper1$^{-/-}$ sperm. No appearance or increase of additional conductance was observed upon heating. Data are averaged from 6 cells. Insert shows representative recordings of residual conductance found in CatSper1$^{-/-}$ sperm. **b** Identical I_{Cs+} densities (pA/pF) presented in (a) on a larger scale. **c** Averaged capacitance (Cm) of CatSper1$^{+/+}$ sperm was similar to Cm of CatSper1-null sperm: Cm $_{\mathrm{CatSper1+/+}}$ = 2.45±0.01 pF, n= 109, and Cm$_{\mathrm{CatSper1-/-}}$ = 2.56 ± 0.03 pF, n= 13. **d** Representative current from CatSper1$^{-/-}$ sperm stimulated by the voltage ramps at 22°C in the presence of different extracellular monovalent ions. Insert shows the main conducting ion and pH of the solutions used. **e** Residual CatSper I_{Cs+} densities (pA/pF) obtained from voltage ramps in the presence of 10 µM NNC 55-0396, a known blocker of CatSper that resulted in 80% CatSper inhibition. No significant increase of I_{Cs+} was observed after heat exposure. Data are averaged from 3 cells. **f-g** Time-course of I_{Cs+} response (upper panels) to heat ramps (lower panels) recorded at -40 mV and -80 mV from CatSper1$^{+/+}$ (left, n=5) and CatSper1$^{-/-}$ (right, n = 4) murine sperm. Insert shows the main conducting ion and pH of the solutions used for (a-c, and e-g).

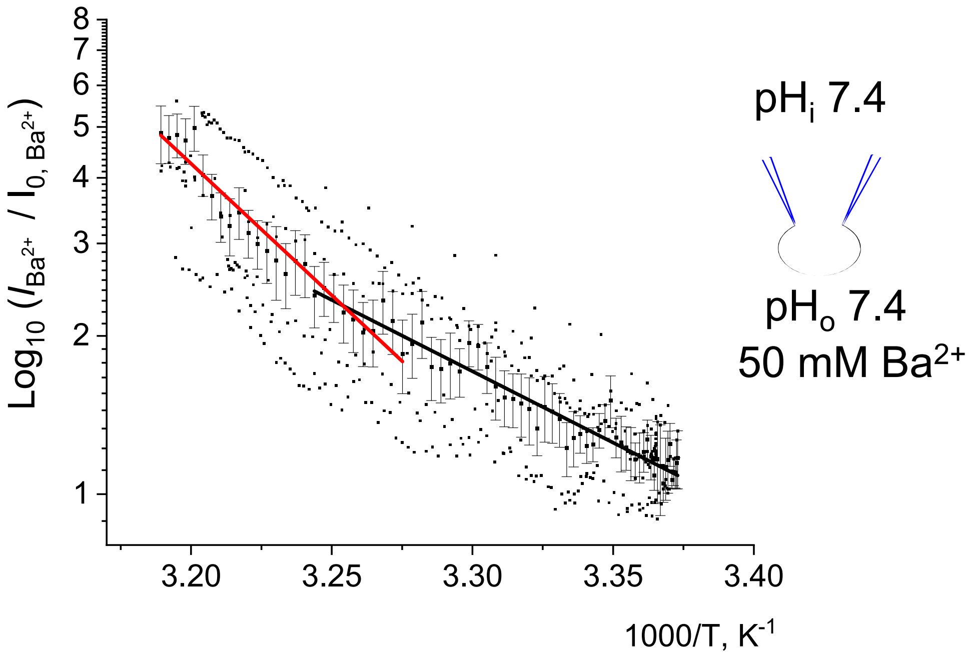

Supplementary Fig. 2 Murine epididymal CatSper is gated by heat. The current-temperature relationship shown in Fig. 2f with individual data distribution. Insert shows the main conducting ion and pH of the solutions used. Data are mean values ± S.E.M. from 7 individual cells.

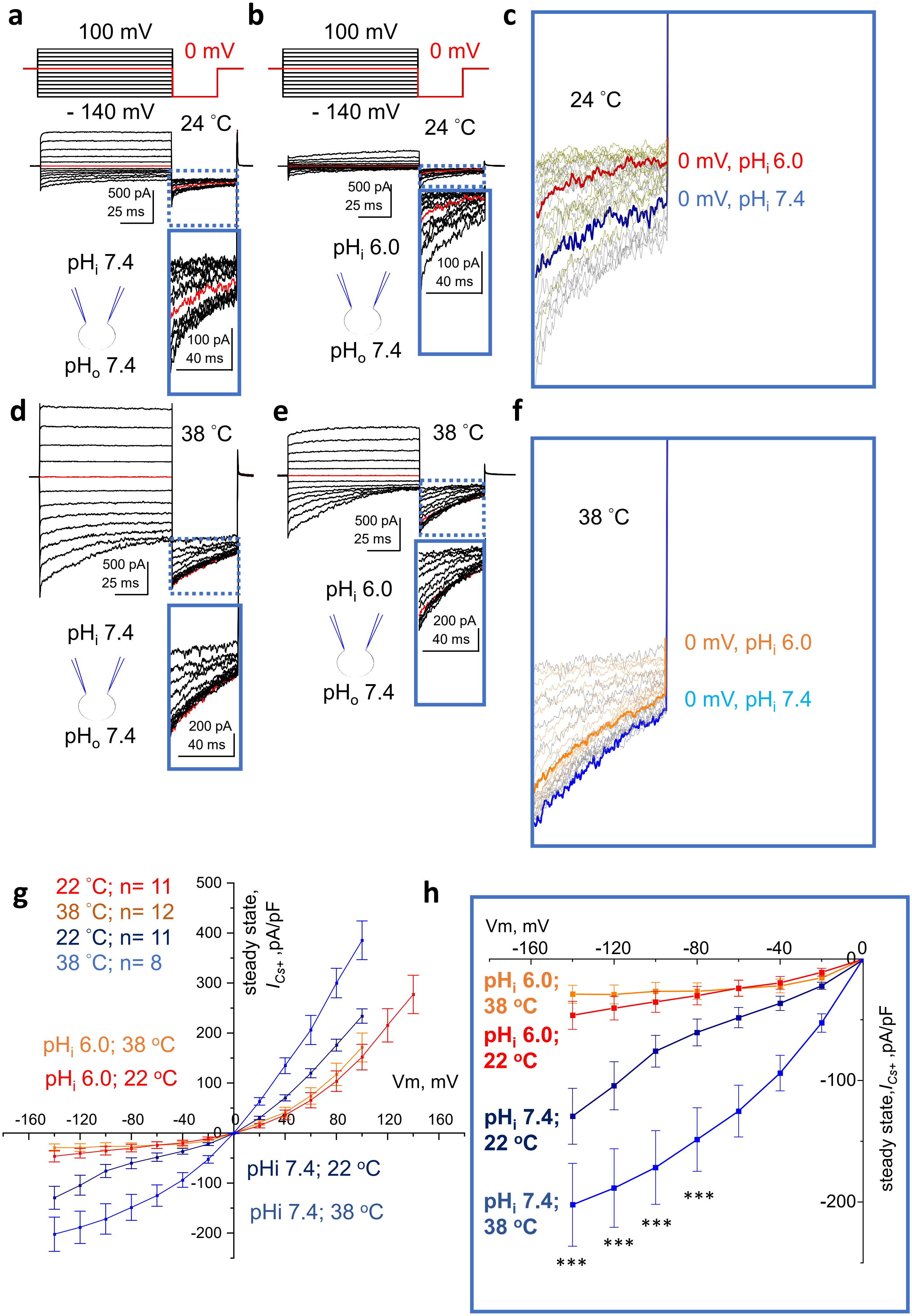

a
100 mV
0 mV
- 140 mV
24 °C
500 pA
25 ms
pHi 7.4
pHo 7.4
100 pA
40 ms
b
100 mV
0 mV
- 140 mV
24 °C
500 pA
25 ms
pHi 6.0
pHo 7.4
100 pA
40 ms
c
24 °C
0 mV, pHi 6.0
0 mV, pHi 7.4
d
38 °C
500 pA
25 ms
pHi 7.4
pHo 7.4
200 pA
40 ms
e
38 °C
500 pA
25 ms
pHi 6.0
pHo 7.4
200 pA
40 ms
f
38 °C
0 mV, pHi 6.0
0 mV, pHi 7.4
g
22 °C; n= 11
38 °C; n= 12
22 °C; n= 11
38 °C; n= 8
pHi 6.0; 38 °C
pHi 6.0; 22 °C
pHi 7.4; 22 °C
pHi 7.4; 38 °C
steady state, ICs+ ,pA/pF
Vm, mV
h
Vm, mV
pHi 6.0; 38 °C
pHi 6.0; 22 °C
pHi 7.4; 22 °C
pHi 7.4; 38 °C
steady state, ICs+ ,pA/pF
***

Supplementary Fig. 3 Intracellular acidic pH inhibits heat activation of CatSper. a-b Representative I_{Cs+} recordings in response to voltage steps at 24°C with intracellular pH 7.4 (left panel) and at intracellular pH 6.0 (right panel). **c** Insert shows overlaid tail currents from (a) and (b). No change in current kinetics is visible. **d-e** Representative I_{Cs+} recordings in response to voltage steps at 38°C with intracellular pH 7.4 (left panel) and at intracellular pH 6.0 (right panel). **f** Insert shows overlaid tail currents from (d) and (e). Like (c) no change in the inactivation kinetics is visible. **g-h** I-V curves were calculated from amplitudes shown in Fig. 1a and Fig. 3a at the steady state (blue triangles). Insert shows the main conducting ion and pH of the solutions used. Data are mean values ± S.E.M, n indicates number of cells included.

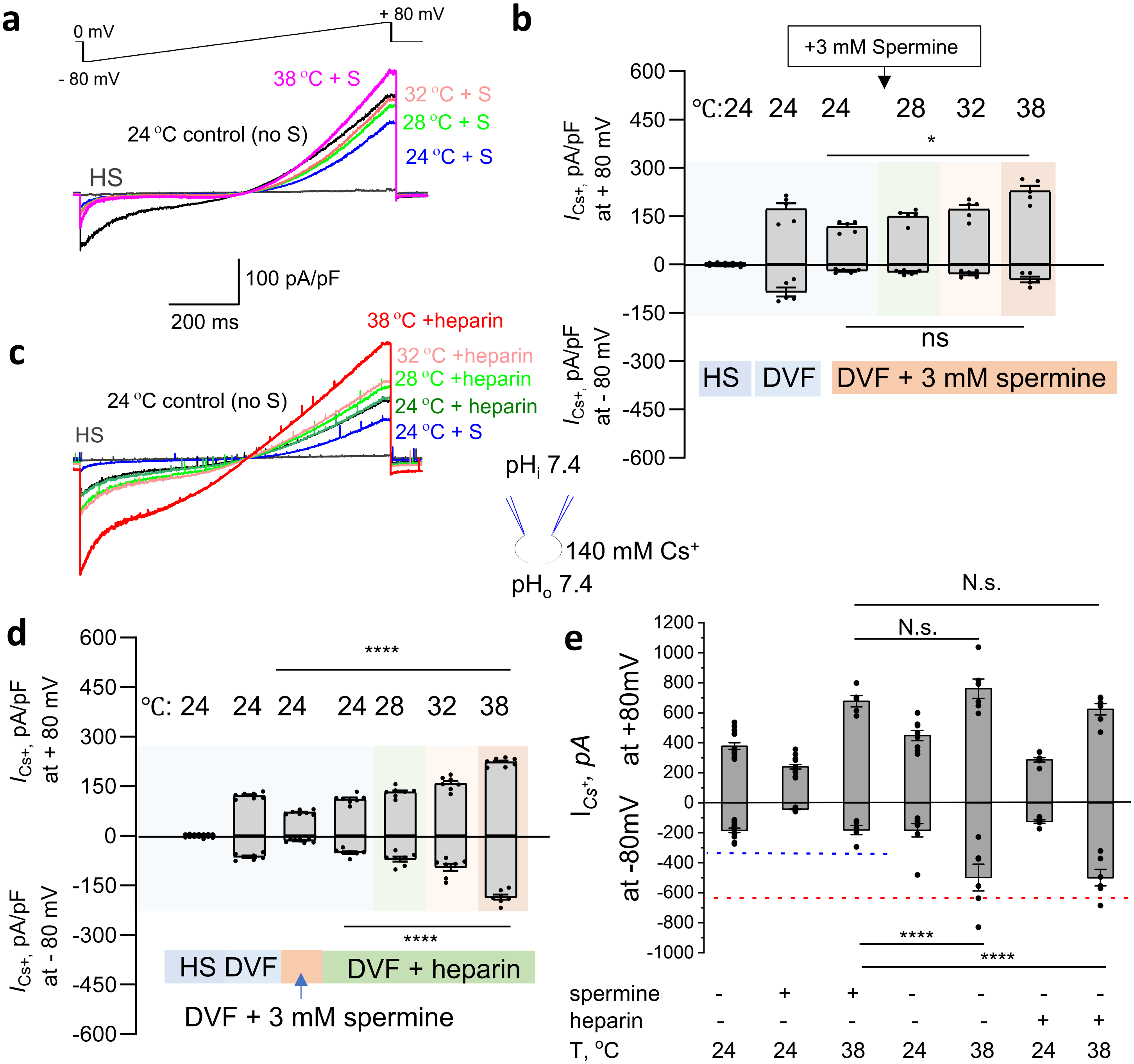

f

	- spermine		+ spermine	
T, °C	24	38	24	38
$V_{1/2}$, mV	-9.4	**-85.5**	-3.7	**25.3**

Supplementary Fig. 4 Spermine reversibly inhibits CatSper. a Representative I_{Cs+} recordings in response to indicated temperatures in the presence of 3 mM spermine (S) and in the absence of spermine (control, no S). **b** I_{Cs+} densities measured at -80 mV and +80 mV as shown in Fig. 4a,d at 24°C, 28°C, 32°C, and at 38°C in the absence and presence of spermine. Data are averaged from 5 cells. **c** Representative I_{Cs+} recordings in response to indicated temperatures in the presence of 3 mM spermine and after spermine removal with heparin. **d** I_{Cs+} densities measured at -80 mV and +80 mV as shown in Fig. 4b,e at 24°C, 28°C, 32°C, and at 38°C after treatment with heparin, restoration of heat response is noticeable. Data are averaged from 3-10 cells. **e** Averaged data from experiments shown on Fig. 4a-b and Figure 1a calculated from I_{Cs+} amplitudes measured at -80 mV and +80 mV. Between 3 to 9 cells were used. Insert shows the main conducting ion and pH of the solutions for this Figure. Data are mean values ± S.E.M., n corresponds to the number of cells used. **f** Table shows midpoint activations for CatSper at indicated conditions.

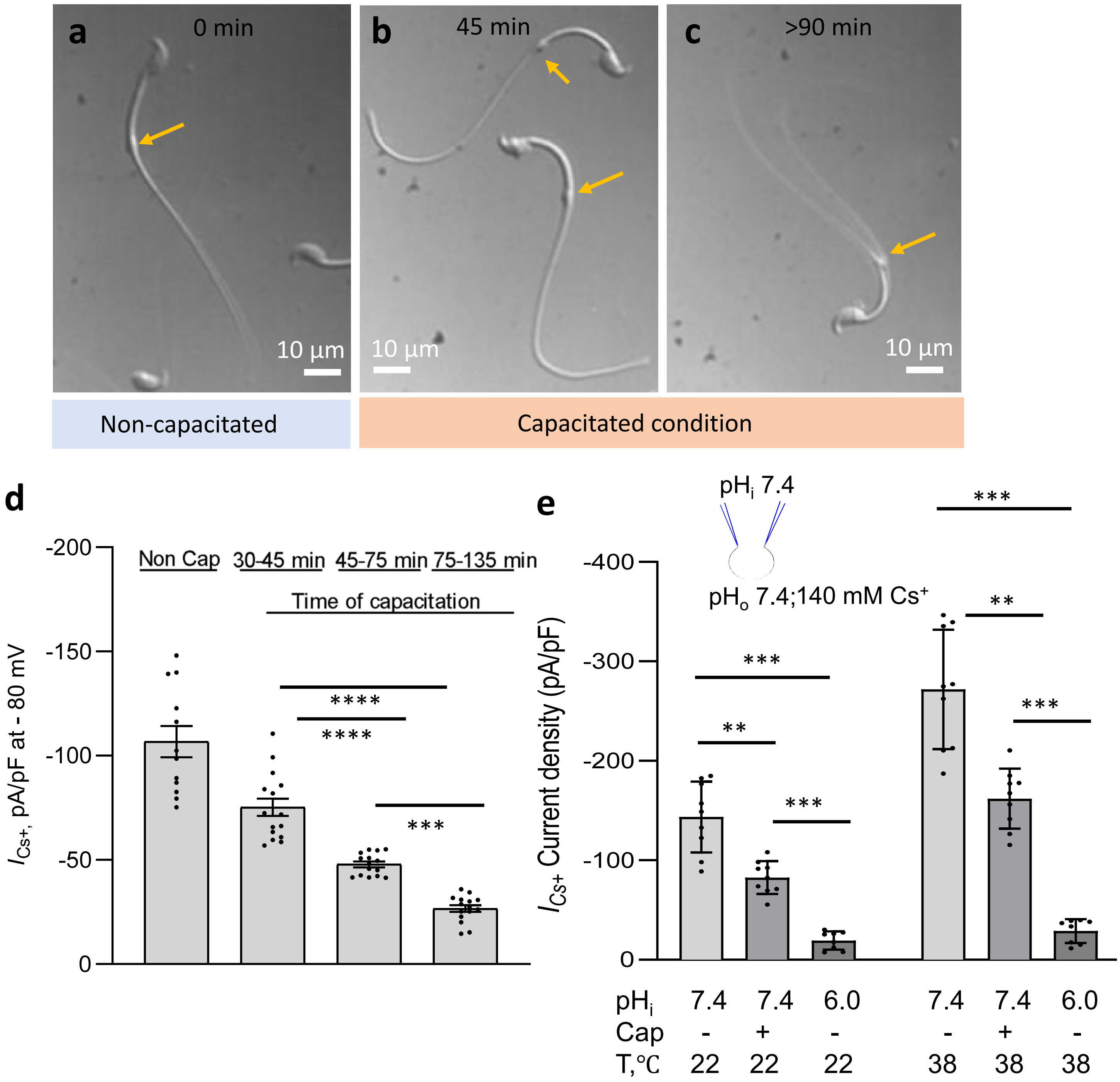

Supplementary Fig. 5 Sperm capacitation and heat response. a-c Differential interference contrast microscopy (DIC) images of representative non-capacitated wild-type murine sperm (a) and capacitated for 45 minutes (b) and 90 minutes (c). Cytoplasmic droplets are indicated by arrows. Note a profound bend of the midpiece regions in (b) and (c) indicating capacitated sperm cells. **d** Time-course of murine sperm capacitation showing a decrease in I_{Cs+} response recorded at 22-24°C as spermatozoa were being kept in capacitating condition; n= 12 for non-capacitated and n=15 per each group of capacitated sperm cells. **e** Averaged I_{Cs+} density recorded at -80 mV from I_{Cs+} evoked by voltage ramps at indicated temperatures, capacitation condition, and pH_i; n= 8-9. Insert shows the main conducting ion and pH of the solutions used in (d) and (e). Data are mean values ± S.E.M.; n corresponds to the number of sperm cells used.

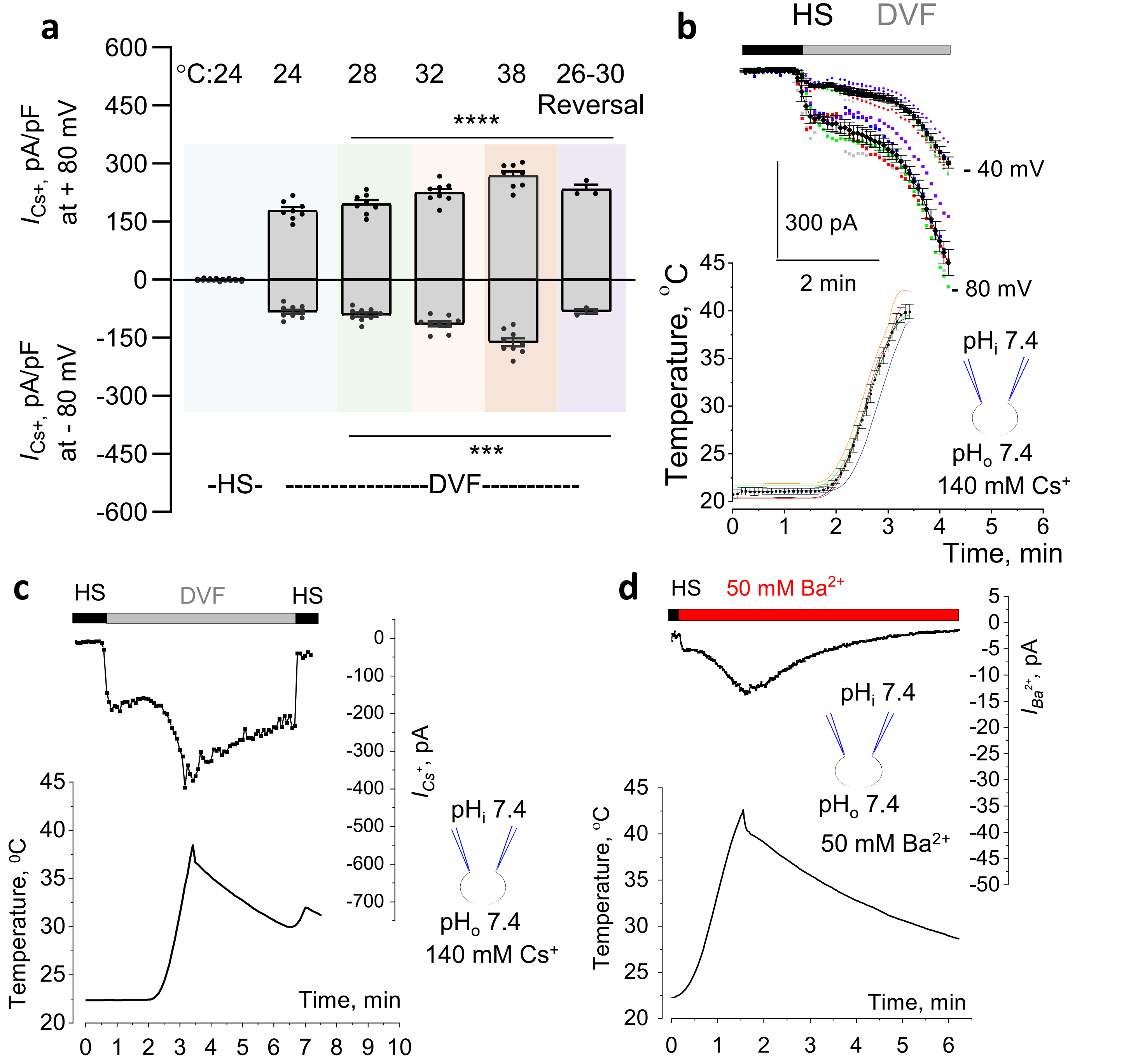

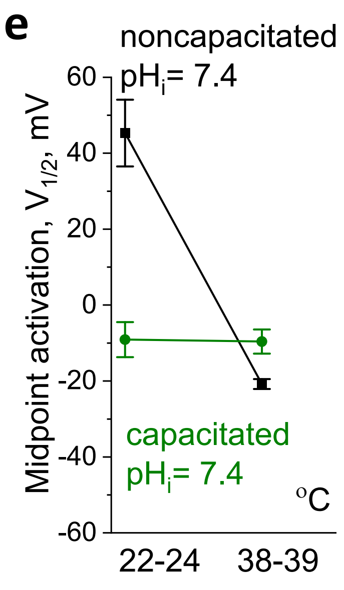

Supplementary Fig. 6 Sperm capacitation and heat response (continue). **a** I_{Cs+} densities from capacitated sperm stimulated by a voltage ramp as shown in Fig. 5a at -80 mV and +80 mV, and obtained at indicated °C. Data are averaged from 3-9 cells. **b** Time-course of I_{Cs+} (upper panel) to heat ramp (lower panel) recorded at -40 mV and -80 mV from capacitated CatSper1$^{+/+}$ sperm (n = 5). **c** Representative heat response of I_{Cs+} recorded from wild-type sperm subjected to capacitation was stable and reversible. Insert shows the main conducting ion and pH of the solutions used in (a-c). **d** Representative heat response of I_{Ba2+} from CatSper1$^{+/+}$ sperm incubated as in (c) was stable and reversible. Insert shows the main conducting ion and pH of the solutions used in (d-e). **e** Data from Fig. 1g (black) and Fig. 5j (green) were used to build a midpoint activation ($V_{1/2}$) plot. No shift in $V_{1/2}$ was observed at 38°C for I_{Ba2+} subjected to capacitating conditions. The absence of significant shift: -9.1 mV at 22°C vs -9.6 mV from at 38°C suggests the adaptive behavior of capacitated CatSper to a warmer oviductal environment. Sperm analyzed for this data set (a-e) were subjected to 45 minutes of capacitation. Data are mean values ± S.E.M.

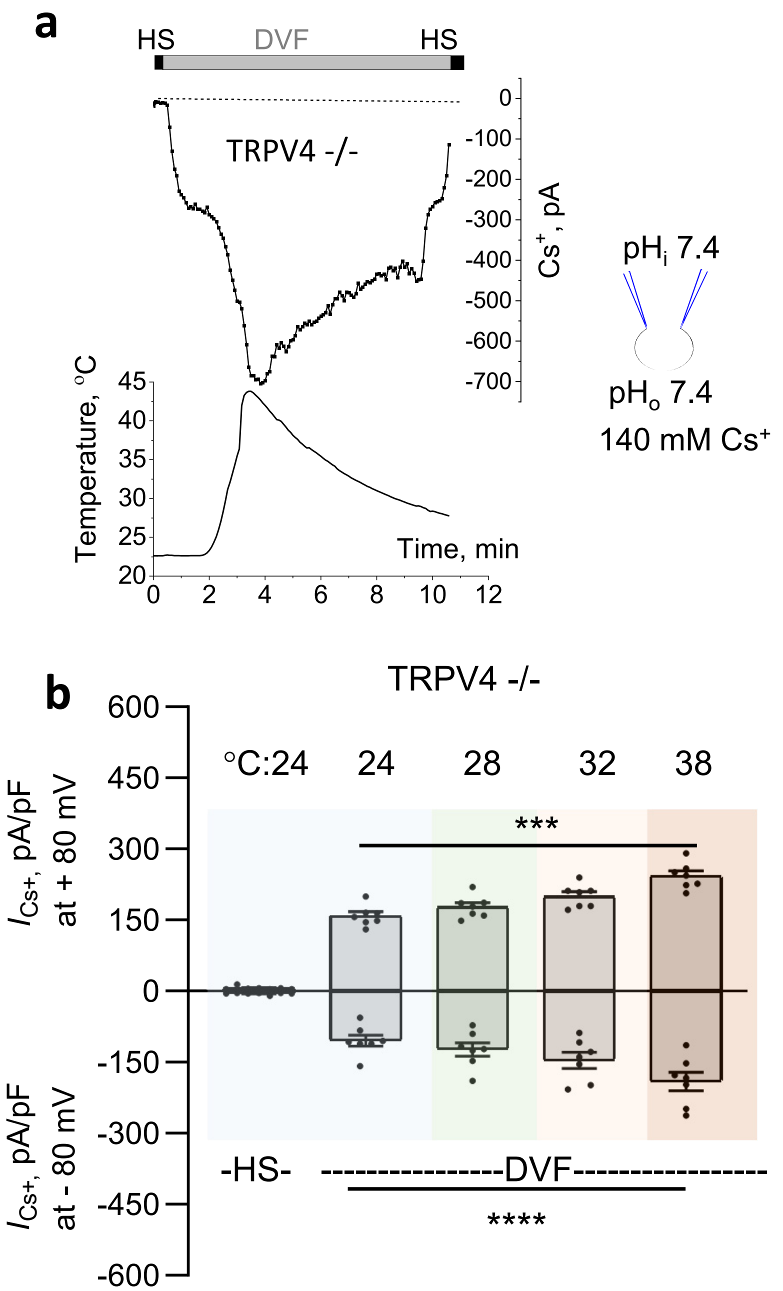

Supplementary Fig. 7 TRPV4$^{-/-}$ murine sperm show similar response to heat as wild-type spermatozoa. a The heat response of I_{Cs+} recorded from TRPV4$^{-/-}$ sperm was stable and reversible. **b** I_{Cs+} densities obtained from TRPV4$^{-/-}$ sperm at -80 mV and +80 mV as shown in Figure 6c (right panel) at 24°C, 28°C, 32°C and at 38°C. Data are averaged from 7 cells. Data are mean values ± S.E.M. Insert shows the main conducting ion and pH of the solutions.

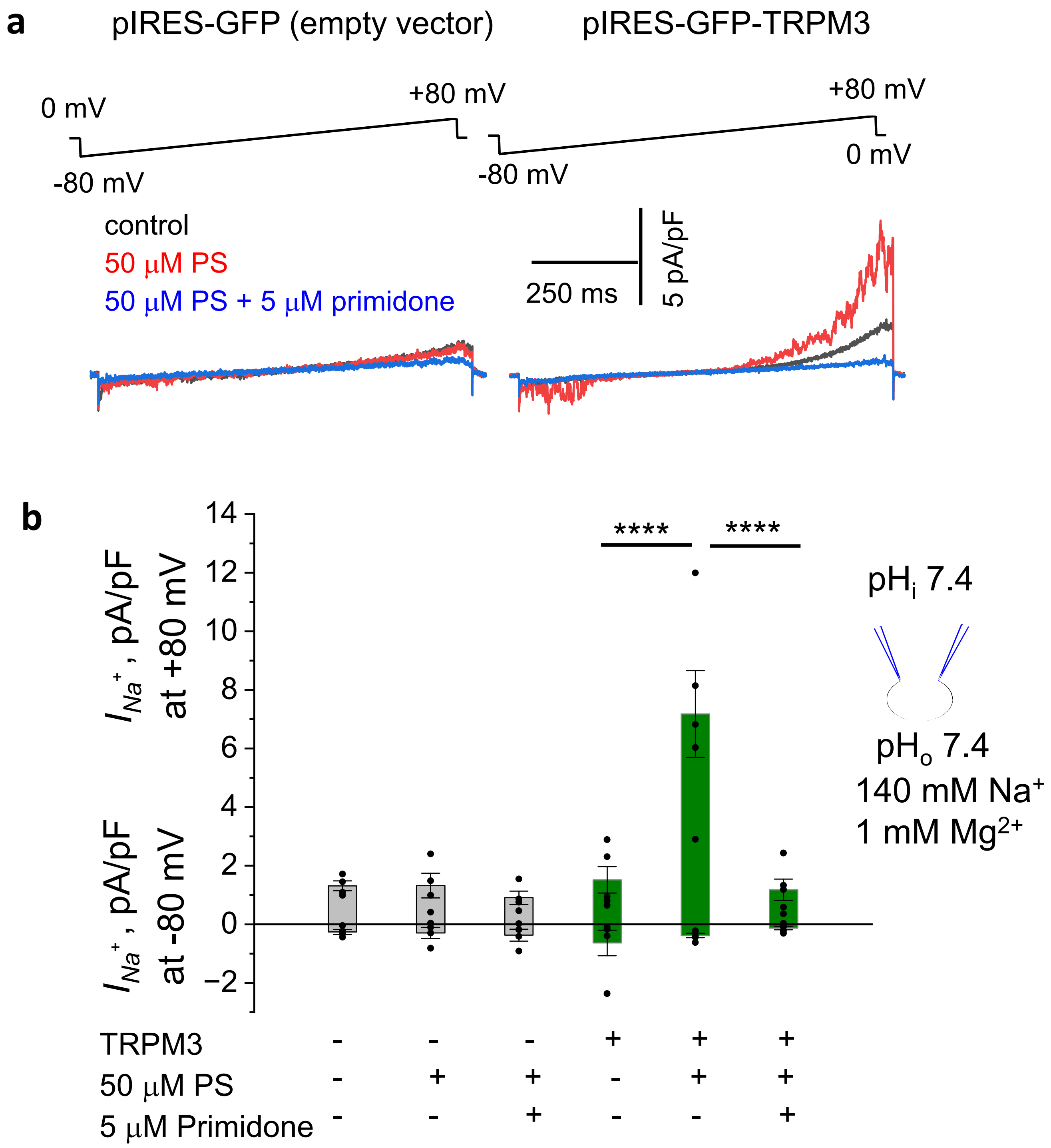

Supplementary Fig. 8 Human TRPM3 expressed in HEK293 cells is sensitive to pregnenolone sulfate and primidone. a Representative I_{Na+} recordings in response to a voltage ramp recorded at room temperatures from HEK293 cells transiently transfected with either empty vector (left panel, control cell) or human TRPM3-containing expression vector pIRES-GFP (right panel). Addition of 50 µM pregnenolone sulfate (PS) did not elicit any response in control cells but produced a strong response in TRPM3-transfected cells (red trace) indicating the presence of the functional channel. This effect was inhibited by TRPM3 antagonist primidone (blue trace). **b** I_{Na+} densities obtained from recordings shown in (a) and sampled at -80 mV and + 80 mV show that both PS and primidone were active as they affected TRPM3-expressing cells (green). Data are mean values ± S.E.M., n= 3-5. Insert shows the main conducting ion and pH of the solutions used.
