# Supplementary material for: The essential calcium channel of sperm CatSper is temperature-gated
Source: Nat Commun. 2025 Apr 17;16:3657. doi: 10.1038/s41467-025-58824-0 (PMC12006431; doi:10.1038/s41467-025-58824-0)
Supplement: Supplementary file 3 — Description of Additional Supplementary Files [file 41467_2025_58824_MOESM3_ESM.pdf]

Description of Additional Supplementary Files

File Name: Supplementary Movie 1
Description: Non-capacitated spermatozoon from CatSper1$^{+/+}$ mouse showing the typical symmetric flagellar beating of the principal piece and rigid midpiece (the neck region). The speed of recording was 500 frames per second (fps), and video recordings were slowed down to playback at 100 fps.

File Name: Supplementary Movie 2
Description: CatSper1$^{+/+}$ murine spermatozoa after 45-minute capacitation. Sperm cells show asymmetric flagellar beating, significant bending of the neck (midpiece), and fast beating amplitude of the flagella with higher lateral displacement. Same recording speed and playback as in Supplementary Movie 1.

File Name: Supplementary Move 3
Description: CatSper1$^{+/+}$ murine spermatozoa after 90-minute capacitation. Sperm cells continue to display asymmetric flagellar beating with higher lateral displacement and infinityshaped movements. Same recording speed and playback as in Supplementary Movie 1.
