# Supplementary material for: The essential calcium channel of sperm CatSper is temperature-gated
Source: Nat Commun. 2025 Apr 17;16:3657. doi: 10.1038/s41467-025-58824-0 (PMC12006431; doi:10.1038/s41467-025-58824-0)
Supplement: Supplementary file 7 — Reporting Summary [file 41467_2025_58824_MOESM7_ESM.pdf]

nature portfolio

Corresponding author(s): Polina Lishko

Last updated by author(s): 3-12-2025

Reporting Summary

Nature Portfolio wishes to improve the reproducibility of the work that we publish. This form provides structure for consistency and transparency in reporting. For further information on Nature Portfolio policies, see our Editorial Policies and the Editorial Policy Checklist.

Statistics

For all statistical analyses, confirm that the following items are present in the figure legend, table legend, main text, or Methods section.

n/a	Confirmed	
☐	☒	The exact sample size (*n*) for each experimental group/condition, given as a discrete number and unit of measurement
☐	☒	A statement on whether measurements were taken from distinct samples or whether the same sample was measured repeatedly
☐	☒	The statistical test(s) used AND whether they are one- or two-sided<br>*Only common tests should be described solely by name; describe more complex techniques in the Methods section.*
☐	☒	A description of all covariates tested
☐	☒	A description of any assumptions or corrections, such as tests of normality and adjustment for multiple comparisons
☐	☒	A full description of the statistical parameters including central tendency (e.g. means) or other basic estimates (e.g. regression coefficient) AND variation (e.g. standard deviation) or associated estimates of uncertainty (e.g. confidence intervals)
☐	☒	For null hypothesis testing, the test statistic (e.g. *F*, *t*, *r*) with confidence intervals, effect sizes, degrees of freedom and *P* value noted<br>*Give P values as exact values whenever suitable.*
☒	☐	For Bayesian analysis, information on the choice of priors and Markov chain Monte Carlo settings
☒	☐	For hierarchical and complex designs, identification of the appropriate level for tests and full reporting of outcomes
☒	☐	Estimates of effect sizes (e.g. Cohen's *d*, Pearson's *r*), indicating how they were calculated

Our web collection on statistics for biologists contains articles on many of the points above.

Software and code

Policy information about availability of computer code

Data collection	n/a
Data analysis	Data were analyzed using Clampfit 10.7, Origin Pro 9.0, Origin Pro2024b, and Prism 8.0.

For manuscripts utilizing custom algorithms or software that are central to the research but not yet described in published literature, software must be made available to editors and reviewers. We strongly encourage code deposition in a community repository (e.g. GitHub). See the Nature Portfolio guidelines for submitting code & software for further information.

Data

Policy information about availability of data

All manuscripts must include a data availability statement. This statement should provide the following information, where applicable:

- Accession codes, unique identifiers, or web links for publicly available datasets
- A description of any restrictions on data availability
- For clinical datasets or third party data, please ensure that the statement adheres to our policy

All data are available in the manuscript or the supplementary materials. The data and materials used in the analysis will be available to any researcher for purposes of reproducing or extending the analysis. RNA-seq data has been submitted to GEO. GEO accession number is GSE290696.

Research involving human participants, their data, or biological material

Policy information about studies with human participants or human data. See also policy information about sex, gender (identity/presentation), and sexual orientation and race, ethnicity and racism.

Reporting on sex and gender	n.a
Reporting on race, ethnicity, or other socially relevant groupings	*Please specify the socially constructed or socially relevant categorization variable(s) used in your manuscript and explain why they were used. Please note that such variables should not be used as proxies for other socially constructed/relevant variables (for example, race or ethnicity should not be used as a proxy for socioeconomic status).* *Provide clear definitions of the relevant terms used, how they were provided (by the participants/respondents, the researchers, or third parties), and the method(s) used to classify people into the different categories (e.g. self-report, census or administrative data, social media data, etc.)* *Please provide details about how you controlled for confounding variables in your analyses.*
Population characteristics	*Describe the covariate-relevant population characteristics of the human research participants (e.g. age, genotypic information, past and current diagnosis and treatment categories). If you filled out the behavioural & social sciences study design questions and have nothing to add here, write "See above."*
Recruitment	*Describe how participants were recruited. Outline any potential self-selection bias or other biases that may be present and how these are likely to impact results.*
Ethics oversight	*Identify the organization(s) that approved the study protocol.*

Note that full information on the approval of the study protocol must also be provided in the manuscript.

Field-specific reporting

Please select the one below that is the best fit for your research. If you are not sure, read the appropriate sections before making your selection.

[x] Life sciences [ ] Behavioural & social sciences [ ] Ecological, evolutionary & environmental sciences

For a reference copy of the document with all sections, see nature.com/documents/nr-reporting-summary-flat.pdf

Life sciences study design

All studies must disclose on these points even when the disclosure is negative.

Sample size	In whole-cell sperm electrophysiology, 5–10 sperm cells from 3–5 mice are typically sufficient for accurate measurements of current (pA) or current density (pA/pF). Throughout the study, we aimed to maintain consistent recordings across sperm cells obtained from different mice. For data collection, we included 5–8 cells, and occasionally 10 or more, from 3–5 mice. This sampling approach demonstrated minimal variation within the group.The approach is supported by power analysis, which provides a strong statistical rationale.
Data exclusions	Most of the recordings obtained during the study were consistent, showing no significant variations among sperm cells from the same mouse or between sperm cells from different mice. However, recordings with higher basal currents caused by leaky currents or unstable seals were excluded, as they produced nonuniform data with large variations.
Replication	Most of the data acquired during the study were reproducible among sperm cells from the same mouse and between those from different mice, allowing us to assess both individual cell effects and inter-mouse variability. Our goal was to replicate any data points that showed variation. Sufficient recordings were obtained from sperm cells of both the same and different mice.
Randomization	This study did not follow the principle of randomization, as our electrophysiological recordings were specific to sperm cells from the same or different mice. This was not a clinical trial or a population-based study but rather a fundamental exploratory investigation. The selected mice were of the same strain and were uniformly maintained under standard conditions. However, random sperm cells were chosen from the population from the same preparation, based on their overall healthy morphology and motility.
Blinding	This was an exploratory study and was not a clinical study. So, we did not follow blinding to avoid the biases in the data obtained

Reporting for specific materials, systems and methods

We require information from authors about some types of materials, experimental systems and methods used in many studies. Here, indicate whether each material, system or method listed is relevant to your study. If you are not sure if a list item applies to your research, read the appropriate section before selecting a response.

Materials & experimental systems

n/a	Involved in the study
☒	☐ Antibodies
☐	☒ Eukaryotic cell lines
☒	☐ Palaeontology and archaeology
☐	☒ Animals and other organisms
☒	☐ Clinical data
☒	☐ Dual use research of concern
☒	☐ Plants

Methods

n/a	Involved in the study
☒	☐ ChIP-seq
☒	☐ Flow cytometry
☒	☐ MRI-based neuroimaging

Eukaryotic cell lines

Policy information about cell lines and Sex and Gender in Research

Cell line source(s)	HEK293, ATCC, CRL-1573
Authentication	directly purchased from vendor (ATCC)
Mycoplasma contamination	vendor confirmed, all reagents and media were purchased from verified vendors and were negative for mycoplasma.
Commonly misidentified lines (See ICLAC register)	n/a

Animals and other research organisms

Policy information about studies involving animals; ARRIVE guidelines recommended for reporting animal research, and Sex and Gender in Research

Laboratory animals	C57BL/6NJ male mice, 3-6 months old, were purchased from Charles River and C57BL/6J mice were purchased from Jackson Laboratory (JAX)
Wild animals	the study did not involve wild animals
Reporting on sex	since the study is about sperm cells, only sperm producing animals (male mice) were used
Field-collected samples	no field collected samples were used
Ethics oversight	Washington University in St. Louis, School of Medicine Animal Care and Use Committee (protocol 22-0251)

Note that full information on the approval of the study protocol must also be provided in the manuscript.

Plants

Seed stocks	no plants were used
Novel plant genotypes	n/a
Authentication	n/a
